# Anti-flavivirus Properties of Lipid-Lowering Drugs

**DOI:** 10.3389/fphys.2021.749770

**Published:** 2021-10-07

**Authors:** Carlos Noe Farfan-Morales, Carlos Daniel Cordero-Rivera, José Manuel Reyes-Ruiz, Arianna M. Hurtado-Monzón, Juan Fidel Osuna-Ramos, Arely M. González-González, Luis Adrián De Jesús-González, Selvin Noé Palacios-Rápalo, Rosa María del Ángel

**Affiliations:** ^1^Department of Infectomics and Molecular Pathogenesis, Center for Research and Advanced Studies (CINVESTAV-IPN), Mexico City, Mexico; ^2^Unidad Médica de Alta Especialidad, Hospital de Especialidades No. 14, Centro Médico Nacional “Adolfo Ruiz Cortines,” Instituto Mexicano del Seguro Social, Heroica Veracruz, Mexico; ^3^Laboratorio de Ingeniería Tisular y Medicina Traslacional, Facultad de Estudios Superiores Iztacala, Universidad Nacional Autónoma de México (UNAM), Mexico City, Mexico

**Keywords:** lipids, flavivirus, antivirals, cholesterol, fatty acids, statins, metformin

## Abstract

Although Flaviviruses such as dengue (DENV) and zika (ZIKV) virus are important human pathogens, an effective vaccine or antiviral treatment against them is not available. Hence, the search for new strategies to control flavivirus infections is essential. Several studies have shown that the host lipid metabolism could be an antiviral target because cholesterol and other lipids are required during the replicative cycle of different Flaviviridae family members. FDA-approved drugs with hypolipidemic effects could be an alternative for treating flavivirus infections. However, a better understanding of the regulation between host lipid metabolism and signaling pathways triggered during these infections is required. The metabolic pathways related to lipid metabolism modified during DENV and ZIKV infection are analyzed in this review. Additionally, the role of lipid-lowering drugs as safe host-targeted antivirals is discussed.

## Introduction

Flaviviruses are a neglected group of human pathogens that cause medically relevant diseases. For example, Zika (ZIKV) and dengue viruses (DENV) are currently relevant health threats in Latin America (San Martín et al., 2010; Ferguson et al., 2016).

After the ZIKV outbreak in the Americas, which left a dramatic increase of microcephaly and brain malformations in newborns, ZIKV disease became an international public health emergency (Ferguson et al., 2016; Schuler-Faccini et al., 2016). Besides the consequences in pregnant women and newborns, the virus also caused an increase in neurological disorders, such as Guillain-Barré syndrome in adults (Cao-Lormeau et al., 2016). Similarly, a considerable rise in DENV cases has been reported in the Americas in recent decades (San Martín et al., 2010). It is estimated that DENV causes 390 million infections per year (Bhatt et al., 2013). Even though many DENV infections resolve without complications, severe dengue is a significant cause of illness and death in some countries in Asia and Latin America (San Martín et al., 2010).

Despite the importance of these pathogens, no specific therapies against DENV or ZIKV are available, and efforts by the scientific community to develop a vaccine or drug for the different flavivirus infections continue (Arredondo-García et al., 2018; Poland et al., 2019). Currently, there is no specific licensed drug to control these viruses, and in most cases, the treatment is palliative with no antiviral effect (Kok, 2016). Therefore, the search for new strategies to help to combat the infections caused by these viruses is essential.

It has recently been documented that the viral cycle of flaviviruses is intimately linked to lipid metabolism (Martín-Acebes et al., 2016b). Specifically, molecules such as cholesterol, which is indispensable during the replication cycle of flaviviruses, are a promising antiviral target (Osuna-Ramos et al., 2018b). Therefore, drugs with hypolipidemic (Lipid-lowering) effects have been proposed as antiviral candidates to treat ZIKV and DENV infections (Osuna-Ramos et al., 2018b; Martín-Acebes et al., 2019). In this review, the metabolic pathways related to lipid metabolism modified during Flavivirus infections are analyzed. Additionally, the role of lipid-lowering drugs as safe host-targeted antivirals is discussed.

## Lipids and the Replicative Cycle of Flaviviruses

Flaviviruses are enveloped viruses of 40–60 nanometers in diameter that belong to the Flaviviridae family. This genus includes more than 50 species of viruses with positive polarity single-stranded RNA of approximately 11,000 nucleotides in length (Barrows et al., 2018).

Flaviviruses depend on lipid metabolism to complete their replication cycle as follows: (a) first, during the viral entry process, the flavivirus envelope lipid bilayers obtained from the endoplasmic reticulum (ER) membrane participate in the viral attachment, binding, and fusion (section “Viral Membrane Composition” and “Flavivirus Entry”); (b) second, an increase in cholesterol and fatty acid synthesis leads to the formation of invaginations of the ER membrane called replicative complexes (RCs) where the viral translation and replication occur (section “Flavivirus Replication”); (c) in the next step, an efficient combination of the cholesterol-rich RCs used as a scaffold and the accumulation of protein C on lipid droplets (LDs) for the viral genome packaging and nucleocapsid formation, contribute to the assembly of the flavivirus progeny (section “Flavivirus Assembly”). Finally, the nucleocapsid buds through the ER membrane completing the virions assembly. The virions are transported through the exocytic pathway to the Golgi complex for its maturation and release from the infected cell.

### Viral Membrane Composition

Although lipids are the most abundant component of the flaviviral particle, with approximately ∼8,000 lipid molecules (Reddy and Sansom, 2016), the composition and biochemistry of the viral envelope have been poorly explored compared to the other components. In general, the membranes of enveloped viruses show a different composition than other cell membranes (Brügger et al., 2006; Kalvodova et al., 2009; Merz et al., 2011; Gerl et al., 2012). Currently, there are no lipidomic analyses of the viral envelope of ZIKV and DENV; however, most of the evidence about the membrane composition of flaviviruses comes from West Nile Virus (WNV) (Martín-Acebes et al., 2014). The WNV envelope has a significant increase in the content of glycerophospholipids (phosphatidylcholine, plasmalogens, and lysophospholipids) and sphingolipids (ceramide, dihydroceramide, and sphingomyelin). Other viruses of the same family, such as hepatitis C virus (HCV) and bovine viral diarrhea virus (BVDV), also showed membranes enriched in sphingolipid and cholesterol (Aizaki et al., 2008; Merz et al., 2011; Callens et al., 2016).

**FIGURE 1:**
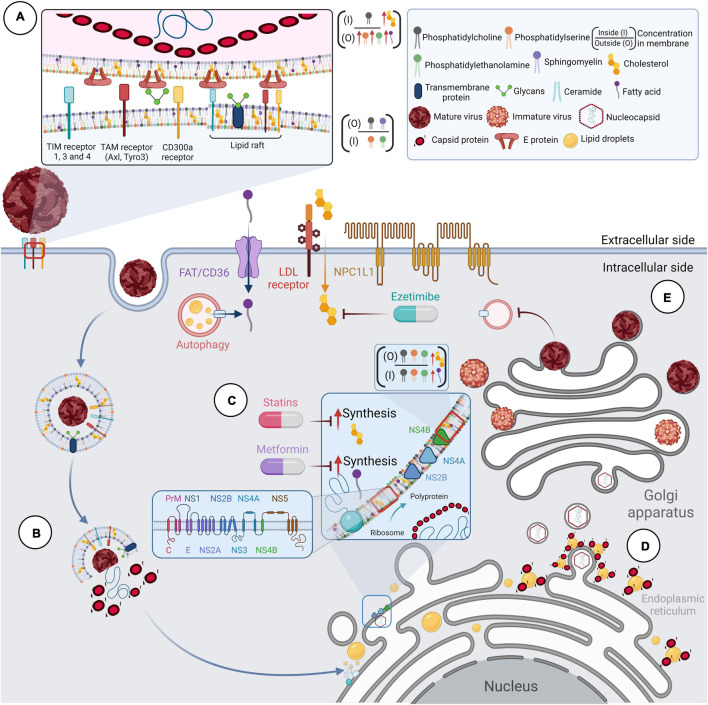
Lipids and the replicative cycle of flavivirus. Flaviviruses depend on lipid metabolism to complete their replication cycle. **(A)** The membranes of Flaviviruses show a different composition than other cell membranes. Lipidomic, computational and functional studies suggest that flavivirus membranes are enriched with glycerophospholipids, sphingolipids, fatty acids, and cholesterol, all of which confer stability and robustness to the virion. During viral entry, contact with receptors allows internalization of the virion into the cell. These cellular receptors are usually coupled to lipid rafts in membranes with well-defined cholesterol concentrations and other membrane-stabilizing elements. **(B)** The viral genome release occurs in late endosomes using compartment-specific lipids. Lipids such as phosphatidylserine and phosphatidylethanolamine are involved in viral binding, entry, and fusion processes. **(C)** The viral RNA is translated into a polyprotein in the ER, which functions as a viral translation, replication, and morphogenesis platform. Infection-induced metabolic *reprogramming* leads to the accumulation of lipids required for viral replication. Lipid requirements are virus-dependent and cell-dependent; however, cholesterol and fatty acids appear necessary for the flavivirus cycle. **(D)** The involvement of lipid droplets (LDs) has been reported during viral replication and assembly. The co-localization and interaction of protein C with LDs have also been described during WNV, DENV, JEV, and ZIKV infections. **(E)** Finally, immature viral particles travel through the Golgi apparatus to complete their maturation process.

Considering that flaviviruses acquire their membrane by sequestering modified fragments of the ER, Reddy and Sansom (2016) analyzed the viral envelope of DENV by computational modeling using lipidomic data from DENV-infected C6/36 cells (Perera et al., 2012; Reddy and Sansom, 2016). This study revealed that the glycerophospholipids, sphingolipids, and fatty acyls are key components of the DENV envelope which could confer stability and robustness to the virion (Reddy and Sansom, 2016). It has been described that specific membrane-enriched lipids (phosphatidylserine and phosphatidylethanolamine) are involved in viral binding, entry, and fusion processes (Figures 1A,B; Meertens et al., 2012; Martín-Acebes et al., 2014; Richard et al., 2015). However, the role of sterols in the viral envelope was not analyzed in the cited studies. The evidence that cholesterol is a component of the flavivirus envelope comes from functional assays (Meertens et al., 2012; Carro and Damonte, 2013; Richard et al., 2015). It has been documented that the amount of sterols in DENV virions is critical since reduction (Carro and Damonte, 2013) and saturation (Lee C. J. et al., 2008) of the cholesterol content in the viral membrane results in loss of infectivity, similar to that reported with the influenza virus (Sun and Whittaker, 2003).

### Flavivirus Entry

#### Viral Binding and Internalization

The first step in the viral replicative cycle is the binding of the virion to the cell surface through one or more receptors that have been proposed for flaviviruses (Cordero-Rivera et al., 2021). Some receptors can interact with lipids and promote viral entry by different mechanisms (van der Schaar et al., 2008; Jemielity et al., 2013; Amara and Mercer, 2015; Cruz-Oliveira et al., 2015; Agrelli et al., 2019). The TIM (1, 3, and 4) (Meertens et al., 2012; Jemielity et al., 2013; Hamel et al., 2015; Richard et al., 2015), TAM (Axl and Tyro3) (Meertens et al., 2012; Bhattacharyya et al., 2013; Hamel et al., 2015), and CD300a receptors (Carnec et al., 2015) can bind to phosphatidylserine and phosphatidylethanolamine on the viral envelope of flaviviruses and contribute to viral internalization (Figure 1A). TIM and TAM receptors could mediate viral internalization by recognizing phosphatidylserine (PS) and phosphatidylethanolamine (PE) on the surface of virions, promoting their entry as apoptotic bodies in a process known as apoptotic mimetics (Meertens et al., 2012; Amara and Mercer, 2015). Interestingly, it has been suggested that the unbalance of calcium during DENV (Dionicio et al., 2018) and YFV (Nour et al., 2013) infection could activate calcium-dependent scramblases that expose PS, to the outer plasma membrane (Figure 1C; Morizono and Chen, 2014).

The participation of cholesterol during viral entry has been related to lipid rafts and microdomains (Figure 1A). Lipid rafts are molecular microdomains located on the plasma membrane, consisting of stable associations between sphingolipids, glycolipids, and cholesterol. They play a crucial role in cellular processes such as signal transduction and membrane protein trafficking (Regen, 2020). These microdomains provide a suitable environment for clustering flavivirus receptors on the host cell and function as platforms for cellular signal transduction (Lee et al., 2005; Reyes-del Valle et al., 2005; Puerta-Guardo et al., 2010; Diwaker et al., 2015). Curiously, cholesterol requirements for lipid raft and microdomain formation appear to be cell-dependent during DENV infection (Lee C. J. et al., 2008; Mosso et al., 2008; Acosta et al., 2009; Rothwell et al., 2009; Carro and Damonte, 2013; Soto-Acosta et al., 2013). Overall, lipid rafts are essential during DENV (Lee C. J. et al., 2008; Puerta-Guardo et al., 2010; Soto-Acosta et al., 2013; García Cordero et al., 2014; Diwaker et al., 2015) and WNV (Medigeshi et al., 2008) entry, while for ZIKV, their relevance is unknown.

#### Membrane Fusion

Following attachment and internalization of the particle, the genome must be released into the cytoplasm by fusion of the viral membrane with that of the late endosomes induced by the low pH of the endosomes (Kaufmann and Rossmann, 2011). It has been documented that DENV ensures its fusion in late endosomes using compartment-specific lipids (Zaitseva et al., 2010), such as other flaviviruses (Figure 1B; Stiasny and Heinz, 2004; Tani et al., 2010; Zaitseva et al., 2010).

Due to the complexity of the process, artificial membranes have been a fundamental tool for studying the importance of lipid composition during the fusion process. For example, Gollins and Porterfield (1986) demonstrated that the lipid composition of liposomes influences both the pH optimum for fusion and the maximum degree of fusion (Gollins and Porterfield, 1986).

It has also been shown that viral fusion can occur even with receptor-free artificial membranes consisting of phosphatidylcholine and phosphatidylethanolamine, and removal of these lipids, including cholesterol, reduces viral fusion (Gollins and Porterfield, 1986; Martín-Acebes et al., 2014).

Regarding the role of cholesterol in membrane fusion, some authors point out that fusion is strongly induced by the presence of cholesterol in the target membrane (Stiasny et al., 2003; Moesker et al., 2010), and others suggest that this molecule is not relevant during this process, and on the contrary, the addition of cholesterol to cells may even reduce flavivirus infection (Lee C. J. et al., 2008; Umashankar et al., 2008). Although more studies are needed to determine the role of cholesterol during the fusion process, it is clear that the lipid composition of the cell and viral membranes are relevant to viral entry and fusion processes.

### Flavivirus Replication

#### Structural Rearrangements of the Endoplasmic Reticulum

Once the viral RNA is released into the host cell cytoplasm, it acts as an mRNA within the infected cell encoding a single open reading frame translated into a polyprotein that undergoes proteolytic cleavage by viral and host proteases. This event produces ten mature proteins, three structural proteins, C (capsid), M (membrane), and E (envelope), and seven non-structural proteins, NS1, NS2A, NS2B, NS3, NS4A, NS4B, and NS5 (Barrows et al., 2018). Although the localization of viral proteins among different cellular compartments is observed during infection (Hannemann et al., 2013; Reyes-Ruiz et al., 2018; Palacios-Rápalo et al., 2021; Zhao et al., 2021), their accumulation is predominant in the ER, the center of lipid synthesis in the cell, which functions as a platform for viral translation, replication, and morphogenesis (Murray et al., 2008). The recently synthesized proteins are anchored to the ER through their transmembrane domain (Barrows et al., 2018), and the interaction between viral proteins and cellular lipids is essential for forming the RCs (Chotiwan et al., 2018). However, membranes remodeling has been mainly associated with the expression of NS2B, NS4A, and NS4B (Figure 1C; Miller et al., 2007; León-Juárez et al., 2016; Leier et al., 2020).

Consequently, while translation and viral replication occur, the ER membrane is remodeled and undergoes considerable enlargement with the appearance of organelle-like structures. These structures function as viral replication factories (Welsch et al., 2009; Peña and Harris, 2012; Junjhon et al., 2014; Hanners et al., 2016; Cortese et al., 2017). Some of these membrane rearrangements may change between flaviviruses and between cell types (Welsch et al., 2009; Junjhon et al., 2014; Hanners et al., 2016; Offerdahl et al., 2017). However, there is a remarkable similarity in the remodeling of intracellular membranes caused by flaviviruses. In this regard, the main structures of the RCs are membrane bundles (Vp), double-membrane vesicles (Ve), tubular structures (T), and convoluted membranes (CM). The localization of NS1, NS3, NS5 proteins, and the dsRNA molecule in Ve suggests that RNA replication occurs in these compartments (Welsch et al., 2009; Junjhon et al., 2014; Cortese et al., 2017).

#### Disturbances in the Lipid Composition of the Endoplasmic Reticulum

Membrane structures resulting from viral replication exhibit a specific lipid composition responsible for the membrane topology in the RCs. Lipid analyses show that certain cellular lipids are modified in flavivirus-infected cells compared to uninfected cells (Figure 1C; Perera et al., 2012; Melo et al., 2016; Chotiwan et al., 2018; Chen et al., 2020; Leier et al., 2020). For example, DENV infection alters approximately 15% of cellular lipids in both C6/36 mosquito cells and the midgut of DENV-infected mosquitoes (Perera et al., 2012; Chotiwan et al., 2018). These modifications are concentrated in the membrane fractions associated with replication, where 85% of the lipid species were significantly modified compared to membranes of uninfected cells (Perera et al., 2012).

The lipidomes of C6/36 cells (Melo et al., 2016), fetal placental cells (Chen et al., 2020), and different human cell lines (Leier et al., 2020) are also changed by ZIKV infection. Similar to DENV, ZIKV perturbed the phospholipid profile and induced increased phosphatidylcholines, phosphatidylethanolamines, and phosphatidylserines in mosquito cells (Perera et al., 2012; Melo et al., 2016). Such alterations were also maintained in placental cells, where phosphatidylinositol was also increased (Chen et al., 2020). In addition, elevated levels of sphingolipids were found in infected C6/36 cells (Melo et al., 2016) and human cell lines (Leier et al., 2020).

Notably, the enrichment of glycerophospholipids and sphingolipids persists in DENV (Chotiwan et al., 2018) and WNV (Martín-Acebes et al., 2014), suggesting that these changes could be maintained in flavivirus infections. However, comparative analyses of ZIKV-infected placentas revealed no alterations in ceramide or sphingolipid subspecies (Chen et al., 2020). Therefore, sphingolipid requirements during ZIKV infection could be variable depending on the cell type.

Regarding cholesterol, a lipidomic study of the intestines of DENV-infected mosquitoes showed that out of 111 sterol molecules detected, 25 showed different levels of abundance compared to controls. Of these, 21 molecules increased, and four molecules decreased during infection. Most of the changes (10 molecules) occurred on day three post-infection, during early infection, and on day seven post-infection (14 molecules), a period of high replication activity in the mosquito middle gut. Only one molecule showed significant changes (decreased) on day 11 post-infection, a period when there is high replication activity in salivary glands and other tissues compared to the middle gut (Chotiwan et al., 2018). Considering that sterols in the mosquito come from the diet, it would be interesting to compare the enrichment of sterols in the lipidome of human and mosquito cells at different times of DENV infection. Unfortunately, there are no other lipidomic studies with DENV and ZIKV where sterols content and related metabolites are analyzed. However, increased cholesterol in Huh-7 cells caused by DENV infection compared to uninfected cells has been reported (Soto-Acosta et al., 2013). The enrichment was concentrated at DENV replication sites, and it was also evident in the first hours of infection. Therefore, it has been suggested that cholesterol is necessary for early stages and during viral replication *in vitro* (Soto-Acosta et al., 2013) and in the *in vivo* mosquito model (Chotiwan et al., 2018).

The ZIKV infection also causes several adaptations in placental lipid metabolism, including increased neutral lipids: cholesterol, diacylglycerols, and triacylglycerols. Metabolic reprogramming consequently triggered the biogenesis of cholesterol-enriched lipid droplets and the intracellular membrane reorganization for viral replication (Chen et al., 2020). This evidence suggests that enrichment of cholesterol and other lipids in infected cells is required during flavivirus infections (Mackenzie et al., 2007; Rothwell et al., 2009; Soto-Acosta et al., 2013); however, how each lipid contributes to viral replication is still being studied (Villareal et al., 2015).

Enrichment of sphingolipids, such as ceramide, during flavivirus infections, is thought to be important for membrane topology (Castro et al., 2014; Villareal et al., 2015), viral budding (Zha et al., 1998; Holopainen et al., 2000; Trajkovic et al., 2008; Hurley et al., 2010), and virion architecture (Martín-Acebes et al., 2014; Reddy and Sansom, 2016). Phospholipids could be involved in membrane fluency and curvature (Roux et al., 2005; Martinez-Seara et al., 2008). Cholesterol in conjunction with ceramide could lead to the formation of microdomains in replication-associated membranes in the ER (Silvius, 2003; Marsh, 2009; Staneva et al., 2009; García-Arribas et al., 2016), working as platforms for viral proteins such as NS3 (García Cordero et al., 2014). Therefore, flaviviruses require a favorable microenvironment with the resources to create their replication platforms and acquire their viral envelope with unique composition and properties.

### Flavivirus Assembly

The viral replication processes and viral assembly are intimately linked; viral particles are produced by budding of nucleocapsids (outgoing genomic RNA-associated protein C) associated with ER-derived membranes containing prM and E proteins. The principal viral proteins involved in virion assembly are NS2A and Capsid (C) protein (Samsa et al., 2009; Teoh et al., 2014; Xie et al., 2019; Zhang et al., 2019; Tan et al., 2020). As mentioned, the viral membrane is derived from modified portions of the ER. Once the membrane is acquired, immature viruses are mobilized along the secretory pathway through the Golgi complex, where prM is processed by the furin protease for maturation and subsequent release from the cell (Barrows et al., 2018).

The involvement of lipid droplets (LDs) has been reported during viral replication and assembly (Figure 1D; Samsa et al., 2009). LDs are cellular organelles that serve as a reservoir of cholesterol and other lipids for membrane formation and maintenance (Walther and Farese, 2012; Olzmann and Carvalho, 2019). These organelles are composed of a neutral lipid core surrounded by a phospholipid monolayer; therefore, they can prevent cellular lipotoxicity by converting excess fatty acids into neutral lipids for storage (Tauchi-Sato et al., 2002). Other functions of LDs have recently emerged, such as avoiding mitochondrial damage during autophagy (Nguyen and Olzmann, 2017) and their involvement in immune responses (Monson et al., 2021).

During flavivirus infection, they function as sites of recruitment of both cellular and viral proteins. For example, it has been described that the NS3 protein of DENV interacts with the Rab18 protein, a small GTPase involved in vesicle trafficking, in LDs to recruit the enzyme fatty acid synthase (FASN) to DENV replication sites and promote fatty acid biosynthesis (Figure 1C; Tang et al., 2014).

The co-localization and interaction of protein C with LDs has also been described during WNV, DENV, JEV, and ZIKV infection (Figure 1D; Samsa et al., 2009; Carvalho et al., 2012; Martins et al., 2012, 2019; Teoh et al., 2014; Shang et al., 2018; Ishida et al., 2019; Saumya et al., 2020). In this regard, dissociation of protein C from LDs inhibits the production of infectious DENV particles, but not RNA replication (Carvalho et al., 2012); therefore, it has been suggested that LDs function as scaffolds for viral genome encapsidation (Samsa et al., 2009).

Furthermore, flaviviruses can use lipids from LDs through lipophagy (Heaton and Randall, 2010) and reabsorption of these organelles (Peña and Harris, 2012). Viruses such as DENV (Samsa et al., 2009; Barletta et al., 2016), ZIKV (Chen et al., 2020), and HCV (Miyanari et al., 2007) manipulate LDs biogenesis to promote viral genome replication and virion production (Cloherty et al., 2020).

Regarding ZIKV, there are different positions; this virus induces large amounts of LDs in infected cells, and these LDs are tangled and accumulate around infected focal sites within infected placental villi to promote viral genome replication (Chen et al., 2020). In contrast, a decrease in the number and volume of LDs in ZIKV-infected Huh-7 cells has also been reported (García et al., 2020). Therefore, the role of LDs during ZIKV infection should be further studied.

## Metabolic Pathways as Therapeutic Targets

### Fatty Acid Biosynthesis

The fatty acid biogenesis appears to be an important therapeutic target against flaviviruses, as it is necessary to synthesize complex lipids such as those enriched during viral infections (Perera et al., 2012; Martín-Acebes et al., 2014; Melo et al., 2016; Chotiwan et al., 2018; Chen et al., 2020; Leier et al., 2020).

There are two key enzymes for fatty acid generation, acetyl-CoA carboxylase (ACC), the limiting enzyme in lipid biosynthesis, and fatty acid synthase (FASN). ACC initially catalyzes the carboxylation of acetyl-Coenzyme A to malonyl-CoA (Tong and Harwood, 2006). In later steps, FASN catalyzes the synthesis of acetyl-CoA palmitate and malonyl-CoA into long-chain saturated fatty acids (Smith et al., 2003).

It has been documented that DENV positively regulates fatty acid synthesis through the interaction of the viral NS3 protein with the FASN enzyme to redirect it to sites of viral replication and stimulate its function (Figure 1C; Heaton et al., 2010).

The ZIKV also increases the expression of the FASN, the fatty acid translocase (FAT/CD36), and the diacylglycerol acyltransferase 1 (DGAT1) (Chen et al., 2020). The transporter FAT/CD36 assists in fatty acids uptake from the exogenous environment, and the ER-resident DGAT1, an essential enzyme for LD biogenesis, catalyzes the final step in triglyceride biosynthesis. Conversely, the inhibition of ACC (Merino-Ramos et al., 2016) or FASN (Heaton et al., 2010; Martín-Acebes et al., 2011; Perera et al., 2012; Poh et al., 2012) reduces flavivirus infection.

In addition to fatty acids, the synthesis of complex lipids, such as sphingolipids, is also required during ZIKV, DENV, and WNV infections (Martín-Acebes et al., 2014, 2016a; Melo et al., 2016; Chotiwan et al., 2018; Leier et al., 2020). Sphingolipid metabolism consists of a complex network of numerous enzymes that are interconnected and regulated at different levels. Nevertheless, at the center of sphingolipid metabolism reside ceramide synthases (CerSs), a group of enzymes that catalyze the formation of ceramides, the precursors of sphingolipids (Mullen et al., 2012).

During ZIKV infection, a marked increase in ceramide levels has been documented by multiple pathways, which redistributes to sites of replication and sensitizes cells for infection (Leier et al., 2020). In contrast, the reduction of ceramide biosynthesis by inhibition of CerS, and the enzyme downstream serine palmitoyltransferase (SPTLC), can inhibit ZIKV and WNV infection (Aktepe et al., 2015; Leier et al., 2020). However, the ZIKV-infected placentas did not reveal any alterations in ceramide or sphingolipid subspecies (Chen et al., 2020), which might suggest that ceramide requirements during ZIKV infection could vary in different cell types.

Ceramides are also enriched in DENV replication-associated membranes (Perera et al., 2012). However, it has been reported that DENV is insensitive to ceramide disruption (Fraser et al., 2014; Carocci et al., 2015); on the contrary, inhibition of CerS and SPTLC enzymes enhances DENV replication (Aktepe et al., 2015). These observations suggest that different flaviviruses have a differential ceramide requirement for replication (Aktepe et al., 2015).

Catabolism of more complex sphingolipids probably contributes to the lipid increase; however, the evidence suggests that flavivirus infections increase the *Novo* biosynthesis of sphingolipids (Perera et al., 2012; Martín-Acebes et al., 2014; Leier et al., 2020). Therefore, the inhibition of fatty acid biosynthesis could be advantageous over inhibition of complex lipids necessary for viral replication, as described in WNV infections (Martín-Acebes et al., 2011), since the latter also contain fatty acids as part of their structure.

### Cholesterol Biosynthesis

The biosynthesis of cholesterol occurs in the ER, but the sterol content in this organelle is low due to the complex regulation of cellular synthesis and transport (Luo J. et al., 2020).

Despite the regulatory mechanisms, active biogenesis and cholesterol accumulation in DENV RCs have been documented (Figure 1C; Mackenzie et al., 2007; Rothwell et al., 2009; Perera et al., 2012; Soto-Acosta et al., 2013). The increase of cholesterol in liver cells and in the mid-intestine of mosquitoes at different time points of DENV infection suggests a dynamic interaction between host cell lipid metabolism and viral replication (Soto-Acosta et al., 2013, 2017; Chotiwan et al., 2018). It has been described that 3-hydroxy-3-methyl-glutaryl-CoA reductase (HMGCR), a key enzyme in the mevalonate pathway that controls the rate of cholesterol biosynthesis, relocalizes to viral replication-associated membranes and is overactivated during DENV and WNV infection (Mackenzie et al., 2007; Soto-Acosta et al., 2013). In addition, positive up-regulation of mevalonate diphosphodecarboxylase (MVD), an enzyme involved in the intermediate steps of the mevalonate pathway, has been reported during DENV infection (Rothwell et al., 2009).

Furthermore, the *Novo* biosynthesis of this lipid is not the only source of sterols; DENV also promotes the uptake of exogenous cholesterol by increasing the expression of the low-density lipoprotein (LDL) receptor (Soto-Acosta et al., 2013) and Niemann-Pick C1-Like 1 receptor (NPC1L1) (Osuna-Ramos et al., 2018a) on the surface of infected liver cells. In mosquito cells, DENV modifies the expression of the LRP-1 protein to prevent cellular cholesterol efflux and thus promote the accumulation of intracellular cholesterol (Tree et al., 2019). Therefore, inhibition of enzymes involved in the mevalonate pathway, such as HMGCR, MVD, squalene synthase (SQS), or 7-dehydrocholesterol reductase (DHCR-7), can reduce flavivirus multiplication (Mackenzie et al., 2007; Rothwell et al., 2009; Martínez-Gutierrez et al., 2011; Peña and Harris, 2012; Soto-Acosta et al., 2013; Españo et al., 2019; Leier et al., 2020). In addition, inhibition of cholesterol import and intracellular trafficking has also been shown to be effective in inhibiting DENV infection (Soto-Acosta et al., 2013; Osuna-Ramos et al., 2018a). Overall, this evidence demonstrates that the mevalonate pathway offers a wide range of potential host-directed therapeutic targets for treating flavivirus infections.

### Autophagy

In addition to anabolic processes, catabolic processes such as autophagy may also contribute to the lipid requirements necessary for viral replication (Lee Y. R. et al., 2008). Autophagy is a cellular homeostatic process involving the formation of autophagosomes for the recycling of damaged cellular proteins and organelles. It should be noted that this mechanism also plays an essential role in the degradation of labeled intracellular pathogens and in the induction of the antiviral response (Lee and Iwasaki, 2008; Deretic et al., 2013). Even though autophagy restricts WNV replication (Shoji-Kawata et al., 2013; Kobayashi et al., 2014), viruses such as DENV and ZIKV have successfully subverted this process to enhance their replication (Lee Y. R. et al., 2008; Chu et al., 2014; Liang et al., 2016; Sahoo et al., 2020).

**FIGURE 2:**
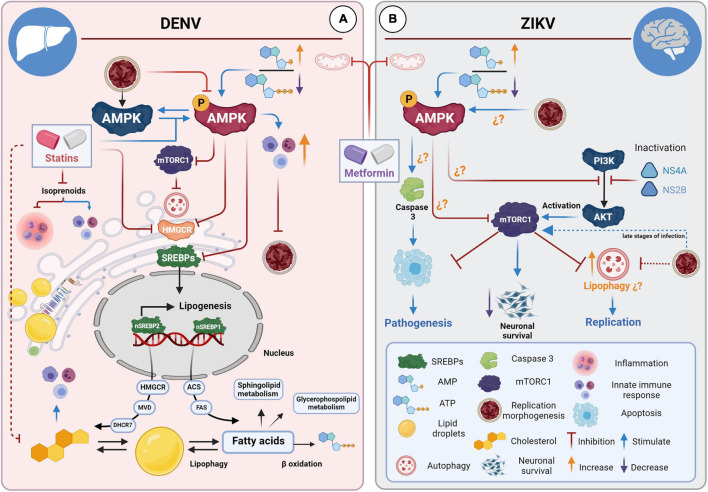
STAs and MET for the treatment of DENV and ZIKV infections. **(A)** STAs and MET inhibit DENV infection in hepatocytes. STAs interfere with cholesterol biosynthesis pathways through competitive inhibition of HMGCR, affecting viral replication, morphogenesis, and progeny during infections in liver cells. STAs enhance the innate immune response by inhibiting isoprenoid synthesis. MET activates the AMPK pathway, which has many downstream targets. MET reduces cholesterol and fatty acid synthesis directly (enzyme inactivation) and via the SREBP pathway. MET also induces the interferon-mediated response via AMPK. Both drugs enhance the innate immune response by connecting the mevalonate pathway and the interferon response in specific cell types. **(B)** MET and neuropathogenesis in the brain of ZIKV-infected mice. It has been suggested that ZIKV can up-or down-regulate cell type-dependent AMPK activity. Activation of AMPK by MET could have a dual effect: counteracting infection in tissues where the virus down-regulates AMPK activity and contributing to the pathology and cell death of tissues where AMPK activation favors viral replication. It has been hypothesized that MET could contribute to apoptosis in neuronal cells.

Autophagy contributes to ZIKV and DENV replication during the early steps of infection by inhibiting apoptosis, evading innate immunity, and altering lipid metabolism for viral replication (Blázquez et al., 2014; Gratton et al., 2019). DENV uses autophagy to degrade LDs and triglycerides to release fatty acids for ATP generation by β-oxidation (Figure 2A; Heaton and Randall, 2010; Heaton et al., 2010). The above is based on a type of selective autophagy called “lipophagy,” in which autophagosomes can target LDs to generate energy for the cell (Singh et al., 2009). In addition, whether DENV can replicate in autophagosomes and double-membrane compartments that are induced during infection remains a controversial question (Khakpoor et al., 2009; Panyasrivanit et al., 2009; Chu et al., 2014).

Although it is not entirely understood how autophagy contributes to viral replication, evidence suggests that autophagy is required during ZIKV and DENV infection. In this regard, autophagy inducers can enhance ZIKV and DENV replication (Hamel et al., 2015; Metz et al., 2015; Liang et al., 2016), while autophagy inhibitors reduce it (McLean et al., 2011; Hamel et al., 2015; Cao et al., 2017). Interestingly, ZIKV-induced autophagy activation has been associated with the early stages of infection. Sahoo et al. (2020) reported that ZIKV induces autophagy early and transiently, and subsequently, the virus can reverse this activation to allow viral protein accumulation and virus replication in neuronal and glial cells; therefore, suppression of autophagy at late times of ZIKV infection is suggested necessary for its replication (Sahoo et al., 2020). Similarly, Metz et al. (2015) reported a biphasic autophagy response to DENV infection, in which DENV infection initially activates it and then, later on, inhibits autophagy (Metz et al., 2015, p. 62).

Although the mechanism is unclear, the evidence suggests that flaviviruses can dynamically modulate autophagy throughout infection. In this sense, it has been demonstrated that ZIKV can induce changes in the activity of the mTORC1 protein (mammalian target of rapamycin complex 1), the master regulator of the autophagic pathway (Sahoo et al., 2020); in turn, mTORC1 can regulate lipogenesis mediated by SREBPs (sterol responsive element binding protein), the transcription factors that regulate cholesterol and fatty acid synthesis (Porstmann et al., 2008; Düvel et al., 2010, p. 1; Li et al., 2010; Peterson et al., 2011, p. 1). Therefore, it cannot be ruled out that ZIKV-induced changes in mTORC1 activity contribute to the switch between lipid catabolism and lipid biogenesis.

Activation of mTORC1 can prevent autophagy following upstream activation of the PI3K/Akt pathway by tyrosine kinase receptors and G protein-coupled receptors in response to their ligands, such as growth factors (LoPiccolo et al., 2008). It has been described that expression of DENV-NS4A can induce PI3K-dependent autophagy and protect epithelial cells against death (McLean et al., 2011); similar, NS4A and NS4B of ZIKV suppress the Akt-mTOR pathway inducing aberrant autophagy in human fetal neural stem cells (fNSCs), leading to defective neurogenesis (Liang et al., 2016). Interestingly, pharmacological inactivation of Akt can also inhibit ZIKV in Vero cells (Albentosa-González et al., 2021, p. 5). Therefore, further studies are required to investigate the therapeutic worth of the PI3K/Akt/mTOR pathway to treat DENV and ZIKV.

## Lipid-Lowering Drugs as Antivirals Candidates

The lack of a vaccine or drug for treating flavivirus diseases has led to an exhaustive search for drugs with anti-flavivirus effects by the scientific community. There are two types of antiviral drugs: the antivirals directed to viral components; and host-targeted antivirals that inhibit key cellular molecules that contribute to the replicative cycle of the viruses (Acosta and Bartenschlager, 2016; Boldescu et al., 2017; Saiz et al., 2018). The drugs that interfere in different metabolic pathways for lipid synthesis belong to the latter group and effectively inhibit flavivirus infections. For instance, drugs that inhibit the enzyme that catalyzes the conversion of sphingomyelin to ceramide, as GW4869, can inhibit the ZIKV infection (Huang et al., 2018); small molecule inhibitors of ACC (PF-05175157, PF-05206574, and PF-06256254) can counteract ZIKV, DENV, and WNV infection (Jiménez de Oya et al., 2019); and the inhibitors of cholesterol synthesis, uptake and transport also have an anti-flavivirus effect (Osuna-Ramos et al., 2018b). Interestingly, this latter group includes FDA-approved drugs such as imipramine (IMI), ezetimibe (EZE), and statins (STAs). The imipramine, an antidepressant, inhibits ZIKV, DENV, and WNV by interfering with intracellular cholesterol transport (Wichit et al., 2017); the EZE inhibits DENV infection by blocking the cholesterol transporter NPC1L1 (Osuna-Ramos et al., 2018a), and STAs have been demonstrated to have a broad anti-flavivirus spectrum by directly inhibiting the HMGCR enzyme responsible for *de Novo* cholesterol synthesis (section “Statins and Ezetimibe for the Treatment of Dengue Viruses and Zika”).

In addition, the drugs that can interfere with both fatty acids and cholesterol synthesis can strongly inhibit flavivirus infection. The SREBP inhibitors, such as Nordihydroguaiaretic Acid and Its derivative Tetra-O-Methyl Nordihydroguaiaretic can inhibit the multiplication of DENV, ZIKV, and WNV, in addition to HCV, suggesting that the SREBP pathway is a therapeutic target (Syed and Siddiqui, 2011; Soto-Acosta et al., 2014; Merino-Ramos et al., 2017). Likewise, the AMP-activated protein kinase (AMPK) activators such as PF-06409577 and AICAR, and Metformin (MET) are effective against DENV, ZIKV, and WNV (Soto-Acosta et al., 2017; Cheng et al., 2018, p. 14–22; Jiménez de Oya et al., 2018). Among the latter group of drugs, MET is another FDA-approved drug with a broad and promising anti-flavivirus spectrum (section “Metformin for the Treatment of Dengue Viruses and Zika”).

FDA-approved drugs with antiviral properties have the advantage of being safe for use in humans (Barrows et al., 2016); this reduces the processes involved in drug development and the administrative and bureaucratic procedures for approval. Therefore, FDA-approved drugs with lipid-lowering effects, such as STAs, EZE, and MET, have been considered as candidate host-directed therapies to treat flavivirus infections (Figure 1; Martín-Acebes et al., 2016b, 2019; Osuna-Ramos et al., 2018b). Although MET is mainly known for its hypoglycemic effects, this drug also can reduce total cholesterol levels and LDL levels in the blood and interfere with fatty acid synthesis (Solymár et al., 2018); therefore, in this article, it is also considered as a candidate lipid-lowering drug to treat flavivirus infection.

### Statins and Ezetimibe for the Treatment of Dengue Viruses and Zika

Cellular cholesterol has emerged as a common denominator among the lipid requirements for flavivirus replication (Osuna-Ramos et al., 2018b). Therefore, inhibition of enzymes that catalyze isoprenoid and cholesterol biosynthesis is effective against DENV (Rothwell et al., 2009; Soto-Acosta et al., 2017). STAs are the drugs of choice for interfering with the cholesterol biosynthetic pathway (Krukemyer and Talbert, 1987). These drugs are structural analogs of HMG-CoA, an intermediate metabolite in the mevalonate pathway, therefore competitively inhibit the HMGCR, the limiting enzyme of this pathway, with an affinity approximately 1,000–10,000 times greater than the natural substrate (Vaziri and Liang, 2004).

Currently, new properties have been revealed in STAs, and a broad spectrum activity to treat diverse human diseases (Pahan, 2006), including viral infections (Gorabi et al., 2020; Wani et al., 2020). *In vitro* assays have shown that DENV is highly susceptible to STAs treatment, which counteracts the over-activation of HMGCR caused by infection (Soto-Acosta et al., 2013). It has been demonstrated that this drug has a strong impact on RCs formation, affecting replication, morphogenesis, and viral yield (Figure 2A; Rothwell et al., 2009; Martínez-Gutierrez et al., 2011; Soto-Acosta et al., 2013; Bryan-Marrugo et al., 2016). In the AG129 immunodeficient mouse model, permissive to flavivirus infection, lovastatin treatment was able to delay mortality of DENV-infected mice by 2 days compared to untreated infected mice (Martinez-Gutierrez et al., 2014).

Currently, only two clinical studies have studied the role of STAs as an antiviral agent for DENV infections. However, no evidence of a beneficial effect on any of the clinical manifestations of DENV or on viremia in adult patients treated with STAs has been found (Whitehorn et al., 2016; Chia et al., 2018). The inability of STAs to inhibit infection is probably related to the concentration needed in liver cells to counteract infection *in vivo*, which is not yet determined. In addition, the reduction of hepatic cellular cholesterol by STAs could be rapidly compensated by LDL-mediated cholesterol import (LDL-Cholesterol) since STAs also positively regulate the LDL receptor and indirectly reduce plasma cholesterol levels (Vaziri and Liang, 2004). Interestingly, there is a correlation between *in vitro* assays, where DENV-infected hepatocytes show an increase of the LDL receptor on the cell surface, and clinical assays, where reduced LDL-cholesterol and total serum cholesterol levels are associated with subsequent risk of developing dengue hemorrhagic fever/dengue shock syndrome (Soto-Acosta et al., 2013; Biswas et al., 2015; Durán et al., 2015). This evidence suggests that exogenous cholesterol uptake plays an important role in DENV replication and pathogenesis.

Therefore, combined pharmacological treatment to inhibit cholesterol biosynthesis and import could be a safe and effective alternative to treat DENV infections.

Drugs such as EZE that selectively inhibit the absorption of cholesterol have also been found effective against DENV *in vitro* infection (Osuna-Ramos et al., 2018a). The target of EZE is the NPC1L1 receptor (Garcia-Calvo et al., 2005); therefore, it acts by blocking the sterol-induced internalization of NPC1L1 (Figure 1; Ge et al., 2008). EZE is usually taken in combination with other lipid-lowering drugs, potentiating the cholesterol-lowering effect (Montecucco et al., 2009; Bach et al., 2019). The combination therapy of STAs with EZE could have a synergistic anti-DENV effect, in addition to the fact that EZE could counteract STAs-induced cholesterol absorption. However, there are currently no studies with combination treatments for flavivirus infections.

Regarding ZIKV, the *in vitro* assays have demonstrated that different STAs effectively inhibit ZIKV replication (Españo et al., 2019; Farfan-Morales et al., 2021). Interestingly only lipophilic STAs showed anti-ZIKV effects, suggesting that lipophilicity is a crucial antiviral property (Españo et al., 2019). In this regard, there is evidence that the lipophilicity of STAs is related to the specificity, efficacy, and pleiotropic effects of these drugs because it allows interaction with lipid membranes (Murphy et al., 2020). However, the role of structure and biophysical properties on the antiviral effects of STAs has been understudied, and there are not *in vivo* studies to confirm that the antiviral properties of these drugs are restricted to lipophilic STAs.

It should be noted that STAs also have several non-cholesterol effects, such as anti-inflammatory and immunomodulatory properties (Gorabi et al., 2020). By inhibiting HMGCR, STAs can inhibit the biosynthesis of isoprenoids that are associated with inflammatory signaling pathways (Ulivieri and Baldari, 2014) and reduce the availability of geranylgeranyl pyrophosphate (GGP) and farnesylpyrophosphate isoprenoids, which are necessary for the prenylation of small G proteins such as Rho and Ras GTPases (Pahan, 2006); these proteins have different functions in intracellular signaling pathways, and some of them participate during the viral replicative cycle (Zamudio-Meza et al., 2009; Wang et al., 2010; Tang et al., 2014; Cuartas-López et al., 2018; Fan et al., 2020).

Furthermore, it has been demonstrated that STAs can also affect the AMPK pathway. As will be described later, AMPK is a therapeutic target for treating flavivirus infections; therefore, the STAs-associated AMPK activation could contribute to its antiviral properties (Dehnavi et al., 2021). In this regard, the effect of STAs on AMPK during flavivirus infections *in vitro* and *in vivo* remains to be elucidated.

Finally, cholesterol metabolism has been linked to the innate and adaptive immune response (Reboldi and Dang, 2018). Studies in macrophages have shown a circuit connecting the cholesterol biosynthetic pathway with the innate immune response (York et al., 2015; Robertson et al., 2016). This suggests that the limitation of cholesterol synthesis and inhibition of the mevalonate pathway by STAs could enhance the immune response in specific cell types. Moreover, inhibition of isoprenoid synthesis and consequent inactivation of small G proteins improves antigen presentation and T cell activation since prenylation of these proteins is required in antigen-presenting cells. In this regard, the decrease lipidation of Rab5 results in arrested endosomal maturation, prolonged antigen retention, enhanced antigen presentation, and T cell activation (Xia et al., 2018). Therefore, the use of STAs as adjuvants has been suggested to increase the efficacy of vaccines against infectious and non-infectious diseases (Xia et al., 2018).

Interestingly, evidence shows that inhibition of the AVM pathway by drugs such as STAs results in immunosuppressive effects (Pahan, 2006). For instance, it has been described that STAs have an inhibitory effect on the proliferation and activation of lymphocytes (Chakrabarti and Engleman, 1991; Shimabukuro-Vornhagen et al., 2014; Wang et al., 2016; Yang et al., 2016). It is probable that the effects of STAs on the immune response depend on the cellular context. However, a great deal remains to be understood about cholesterol metabolism and its connection with the innate and adaptive immune response to understand the antiviral mechanisms underlying STAs (Gorabi et al., 2020).

### Metformin for the Treatment of Dengue Viruses and Zika

MET, a biguanide derivative, has been the most widely used drug to treat type II diabetes for almost a century (Bailey, 2017). In addition to its hypoglycemic effect (Takashima et al., 2010; An et al., 2020), MET can reduce lipid synthesis by activating AMP-activated protein kinase (AMPK), the master regulator of cellular metabolism (Figure 2A; Zhou et al., 2001; Solymár et al., 2018).

The molecular mechanism of action of MET remains in part unknown; however, it has been suggested that being a cation, it accumulates in the mitochondria due to the electrical gradient of the inner membrane, inhibiting complex I of the mitochondrial respiratory chain (Owen et al., 2000; Fontaine, 2018). MET, therefore, inhibits mitochondrial ATP synthesis and consequently causes indirect activation of AMPK, which is sensitive to ATP depletion (Zhou et al., 2001). AMPK activation gives this drug unique properties so that in recent years, many additional functions have been found for MET. Studies have demonstrated a strong effect of MET on numerous cancers, cardiovascular diseases, liver diseases, obesity, and neurodegenerative diseases (Lv and Guo, 2020). The excellent safety profile and lipid-lowering properties have suggested this drug can treat DENV and ZIKV infection (Figure 2A; Soto-Acosta et al., 2017; Martín-Acebes et al., 2019; Farfan-Morales et al., 2021).

It has been described that DENV infection (Soto-Acosta et al., 2017) can inactivate the AMPK kinase, decreasing its active form, phosphorylated to Thr-172 (pAMPK). The complete repercussions of this alteration are unknown, but it has been suggested that it causes profound metabolic changes to provide a favorable host lipid environment for replication, as the over-activation of the HMGCR enzyme (Soto-Acosta et al., 2017). In contrast, activation of AMPK protein by MET can counteract the cholesterol increase and metabolic changes induced by DENV infection (Soto-Acosta et al., 2017).

The anti-DENV effect of MET has been documented in both *in vitro* and *in vivo* assays (Soto-Acosta et al., 2017; Farfan-Morales et al., 2021). MET treatment significantly increased (2 days) the average survival rate in DENV-infected and treated AG129 mice compared to untreated mice and reduced the severe signs of the disease (Farfan-Morales et al., 2021). It has also been reported an association between the use of MET in diabetic patients and the lower risk of suffering a severe disease caused by DENV (Htun et al., 2018), suggesting that MET treatment could attenuate and/or prevent severe forms of DENV infection. Due to the anti-DENV potential of this drug, it is currently being tested as adjunctive therapy for dengue in overweight and obese patients (Nguyen et al., 2020).

On the other hand, MET treatment effectively inhibits ZIKV replication in different cell lines (Farfan-Morales et al., 2021); MET affected the synthesis and distribution of viral proteins in the RCs and reduced viral progeny and double-membrane structures associated with viral replication. However, the treatment fails to counteract the negative signs of the disease or increase the half-life of ZIKV-infected AG129 mice (Farfan-Morales et al., 2021). This could be explained by the fact that the well-established target tissue of MET is the liver, and therefore, the drug appears to be more effective in inhibiting DENV and not ZIKV infection (Gormsen et al., 2016; Jensen et al., 2016). In addition, MET is not metabolized; instead, it is secreted by the kidneys; its short half-life (1.7–7.3 h) and distribution (1.12 ± 0.08 L/kg) suggests a low accumulation in other tissues compared to the liver (Pentikäinen et al., 1979; Scheen, 1996; Shu et al., 2008).

Another explanation could derive from the up-or down-regulation of AMPK activity during ZIKV infection. The evidence suggests that ZIKV may differentially modulate AMPK activity in specific cell types. Specifically, MET could have a dual effect: counteracting infection in tissues where the virus down-regulates AMPK activity and contributing to the pathology and cell death of tissues where AMPK activation favors viral replication (Figure 2B; Thaker et al., 2019). It should be noted that the consequences of AMPK activation in neuronal tissue remain controversial (Ronnett et al., 2009). It has been suggested that excessive and sustained activation of neuronal AMPK under conditions of metabolic stress may lead to neuronal death (Garcia-Gil et al., 2003; McCullough et al., 2005; Chen et al., 2009; Ronnett et al., 2009). Likewise, although MET prevents oxidative stress-induced cell death, it can also induce cell death under certain conditions (Fontaine, 2018). Therefore, considering the increase of pAMPK in brain tissue of Infar1^–/–^ mice and the aggravation of neurological signs in ZIKV-infected female AG129 mice during MET treatment (Farfan-Morales et al., 2021), it cannot be ruled out the possibility that MET may contribute to cell death in specific tissues, as suggested by Thaker et al. (2019).

On the other hand, it is unknown whether autophagy induction and ZIKV-induced AMPK activation in neuronal tissue (Thaker et al., 2019) are co-dependent events. Therefore, despite the pleiotropic effects offered by MET through AMPK activation, the use of this drug to treat ZIKV infections could be disadvantageous due to the multiple target organs it infects. However, it remains to be determined whether the modulation of AMPK by ZIKV is cell-dependent and if there is a causal association between AMPK activity and ZIKV-induced pathology in neuronal lineage cells (Figure 2B).

In contrast, the pleiotropic effects of MET through AMPK can provide a robust response against DENV. In this context, MET, in addition, to directly inactivating key enzymes of different metabolic pathways, such as HMGR and ACC, can also inhibit the different isoforms of SREBPs and, therefore, the expression of genes related to the biogenesis of cholesterol, fatty acids, and triglycerides (Figure 2A; Ha et al., 1994; Zhou et al., 2001). Moreover, it is well known that there is a close connection between AMPK and mTORC1 (Kim et al., 2011); therefore, further studies are required to elucidate the relationship between autophagy and AMPK during flavivirus infections to determine the role of MET during these processes.

Finally, MET may contribute to a more robust immune response through the inflammatory circuitry that joins the regulation of the sterol pathway with the antiviral interferons (IFNs) defense responses (Blanc et al., 2011; York et al., 2015). MET can also enhance the innate immune response through AMPK activation and consequently induce the expression of type I interferon genes in human endothelial cells and hepatocarcinoma cells during ZIKV and HCV infection, respectively (Prantner et al., 2017; Tsai et al., 2017; Singh et al., 2020). Moreover, it has been reported that MET activates the STING/IRF3/IFN-β pathway by inhibiting AKT phosphorylation in pancreatic cancer (Ren et al., 2020). Therefore, in addition to limiting energy and lipid resources, this drug could enhance the innate immune response mediated by type I IFNs.

### Lipid-Lowering Drugs for Other Viral Infections

As described so far, reprogramming of cellular lipid metabolism is virus-dependent; consequently, the susceptibility to lipid-lowering drugs will depend on the requirements for each virus. Although viral lipidomes and the role of lipids in the viral cycle remain underexplored areas, functional studies have shown that many enveloped viruses are susceptible to lipid-lowering drugs (Table 1), such as STAs, which have been suggested to treat infections caused by HCV, Japanese encephalitis virus (JEV), Influenza A virus (IAV), and the Severe acute respiratory syndrome coronavirus 2 (SARS-CoV-2) (Castiglione et al., 2020; Gorabi et al., 2020; Wani et al., 2020).

**TABLE 1:** Study of the antiviral effect of FDA-approved drugs, STAs, EZE, and MET.

Lipid-lowering drug	Virus	Study type	Effect	References
LOV and FLV	DENV	*In vitro*: Human Peripheral blood mononuclear cells (PBMC) and A459 cells.	Inhibition of viral replication.	Rothwell et al., 2009
LOV	DENV	*In vitro*: Human endothelial cell line HMEC and Vero cells	Inhibition of viral assembly.	Martínez-Gutierrez et al., 2011
LOV and PRV	DENV	*In vitro*: Huh-7 cells	Reduction of virus yield and viral RNA transcripts.	Soto-Acosta et al., 2013
LOV	DENV	*In vivo*: AG129 Mice	Delayed infection and increased survival rate.	Martinez-Gutierrez et al., 2014
FLV, ATV, LOV, PRV and SIM	DENV	*In vitro*: Huh-7 cells	Reduction of viral yield by modulation of the cellular antiviral profile.	Bryan-Marrugo et al., 2016
LOV	DENV	Randomized, Double-Blind, Placebo-Controlled Trial: 300 Vietnamese adults with a positive dengue NS1.	No evidence of a beneficial effect of statins on any of the clinical manifestations or on dengue viremia.	Whitehorn et al., 2016
LOV	DENV	*In vitro*: Huh-7 cells	Disruption in the formation of replicative complexes.	Soto-Acosta et al., 2017
SIM, LOV, RSV, and PRV	DENV	Retrospective cohort study: 257 adult dengue patients with hyperlipidemia.	Statin use was not associated with a lower risk of FHD/DSS.	Chia et al., 2018
ATV, CRV, FLV, LOV, MEV, and SIM	ZIKV	*In vitro*: Vero cells	Reduction of virus yield.	Españo et al., 2019
LOV	ZIKV	*In vitro*: Huh-7 cells	Reduction of infected cells.	Farfan-Morales et al., 2021
ATV	JEV	*In vitro*: Neurosphere culture from SVZ region from BALB/c mouse pup brains *In vivo*: BALB/c mouse	*In vitro*: reduction of cell death. *In vivo*: Reduction of viral load in the SVZ. Inhibition of microglial activation and proinflammatory cyto/chemokine production.	Wani et al., 2020
LOV	RSV	*In vitro*: HEp-2 cells *In vivo*: C57BL/6 or BALB/c mice	*In vitro*: Reduction of viral replication. *In vivo*: Reduction of viral replication and virus-induced illness score in mice.	Gower and Graham, 2001
LOV	RSV	*In vitro*: murine RAW 264.7 (RAW) macrophage cell line and primary murine lung macrophages.	Treatment mitigates the pro-inflammatory cytokine response. Lovastatin treatment did not inhibit RSV infection.	Ravi et al., 2013
FLV	HCV	Clinical Trials: Patients with chronic HCV	Lower HCV RNA titers	Bader et al., 2008; Forde et al., 2009
MEV and PTV.	HCV	*In vitro*: Replicon system in hepatocyte cells.	Reduction of replication	Delang et al., 2009; Moriguchi et al., 2010
PTV	HCV	Retrospective and prospective randomized pilot study: HCV Patients with genotype 1b.	Reduction of viral load and enhancement of the SVR	Shimada et al., 2012; Yokoyama et al., 2014
SIM	HBV	*In vitro*: HepG2.2.15	Inhibition of replication.	Bader and Korba, 2010
ATV	HBV	Case Report	Hepatitis B virus reactivation associated with ATOR	Wu, 2013
FLV	CMV	*In vitro*: HUVECs	Decreased viral DNA concentration, viral particle concentration and replication.	Potena et al., 2004.
LOV, FLV, SIM, ATV, RSV, and PTV	EBOV	*In vitro*: Primary human monocyte-derived macrophages and Huh-7 cell line.	Decreased infection. Reduction of the infectivity of the released viral particles.	Shrivastava-Ranjan et al., 2018
SIM	EBOV	*In vitro*: transfected HeLa cells with FLAG-GPs.	Reduction of EBOV glycoprotein-mediated cytotoxicity.	Hacke et al., 2015.
FLV	IAV (H1N1)	*In vitro*: MDCK and A549 cells	Reduction of viral RNA and proteins. Protects host cells against influenza-induced inflammation.	Peng et al., 2014
LOV/caffeine combination	IAV (H5N1,H3N2 and H1N1)	*In vivo*: BALB/c mice	LOV/caffeine combination effectively ameliorated lung damage and inhibited viral replication	Liu et al., 2009
ATV, CRV, FLV, LOV, PRV, and SIM	IAV (H1N1)	Cohort study over 10 influenza seasons (1996 to 2006) using linked administrative databases in Ontario, Canada.	Reduced risk of mortality	Kwong et al., 2009
STAs (not specified)	IAV and IBV (H1N1)	Retrospective analysis: 526 hospitalized patients from Israel with laboratory-confirmed 2017-2018 influenza.	Use of STAs was not associated with mortality benefit.	Atamna et al., 2019
LOV	HIV-1	*In vivo*: SCID mice grafted with adult human PBMCs Clinical trial: Patients in A1 disease stage	Reduction in viral load and increase in CD4+ T-cell count	del Real et al., 2004
ATV, RSV, SIM, PRV, FLV and PTV	SARS-CoV-2	Retrospective study: 13,981 patients with COVID-19 in Hubei Province, China; 2921 patients COVID-19, who are hospitalized in 150 Spanish hospitals.	Reduced risk of mortality among people with COVID-19	Zhang et al., 2020; Torres-Peña et al., 2021
EZE	HCV	*In vitro*: Huh-7 cells *In vivo*: Chimeric mice, uPA/SCID mice with human hepatocytes.	Inhibition of Infection *In vitro* and *In vivo* EZE potently blocks HCV uptake and delays the establishment of HCV genotype 1b infection in mice with human liver grafts.	Sainz et al., 2012
EZE	HBV	*In vitro*: Differentiated HepaRG cells.	EZE inhibits the establishment of intrahepatic cccDNA and expression of viral replication markers.	Lucifora et al., 2013
EZE	DENV	*In vitro*: Huh-7 cells.	Decreased infected cells, viral yield, viral RNA and protein synthesis. Cholesterol-dependent antiviral effect.	Osuna-Ramos et al., 2018a
MET	DENV	*In vitro*: Huh-7 cells.	Disruption in the formation of replicative complexes. Inhibition of viral yield, protein, and cell infection.	Soto-Acosta et al., 2017
MET	DENV	Retrospective cohort study: 223 DENV patients with diabetes mellitus.	Lower risk of suffering a severe disease caused by DENV.	Htun et al., 2018
MET	DENV	*In vitro*: Huh-7 cells *In vivo*: AG129 mice	*In vitro*: Decreased viral yield, protein, and percentage of infected cells. *In vivo*: Reduced virus-induced illness score in mice and increased survival rate.	Farfan-Morales et al., 2021
MET	ZIKV	*In vitro*: Huh-7 cells *In vivo*: AG129 mice	*In vitro*: Decreased viral yield, protein, and percentage of infected cells. *In vivo*: No evidence of a beneficial effect.	Farfan-Morales et al., 2021
MET	YFV	*In vitro*: Huh-7 cells	*In vitro*: Decreased viral yield, protein, and percentage of infected cells.	Farfan-Morales et al., 2021
MET	HCV	Randomized Controlled Trial: 98 patients with genotype 1 chronic hepatitis C and insulin resistance	The combination of MET, peginterferon alfa-2a, and ribavirin increased the SVR rate of patients with hepatitis C genotype 1, with a good safety profile.	Yu et al., 2012
MET	HCV	*In vitro*: OR6 cells and Huh 7.5.1 cells.	Activation of type I interferon signaling. Reduction of replication via AMPK.	Tsai et al., 2017
MET + SIM combination	HCV	*In vitro*: Huh7.5 cells.	Treatment with both drugs inhibited Huh7.5 cell growth and HCV infection via mTOR.	Del Campo et al., 2018
MET	HBV	*In vitro*: HepG2 and HepG2.2.15 cell line	Moderate inhibition of HBV replication.	Xun et al., 2014
MET	CVB3	*In vitro*: Hela cells and primary myocardial cells	Inhibition of replication by reducing lipid accumulation through suppression of lipid synthesis-associated gene expression.	Xie et al., 2015
MET	KSHV	*In vitro*: primary human umbilical vein endothelial cells *In vivo*: BALB/c mice	*In vitro*: Inhibition of viral replication, viral lytic gene expression and production of infectious virions. *In vivo*: Decreased viral replication and increased survival rates.	Cheng et al., 2016
MET	SARS-COV2	Retrospective studies: 283 Hospitalized diabetic patients with confirmed COVID-19 in the Tongji Hospital of Wuhan, China. 1139 COVID-19 positive patients in 8 states in United States. 775 nursing Home Residents Infected with SARS-CoV2 from the Community Living Centers (CLC), United States.	Antidiabetic treatment with metformin was associated with lower hospitalization and mortality. Relative survival benefit in nursing home residents on metformin.	Luo P. et al., 2020; Ghany et al., 2021; Lally et al., 2021

*DENV, Dengue virus; ZIKV, Zika vírus; JEV, Japanese encephalitis virus; RSV, Respiratory Syncytial Virus; HCV, Hepatitis C virus; HBV, Hepatitis B virus; CMV, cytomegalovirus; EBOV, Ebola virus; IAV, Influenza A virus; IBV Influenza B virus; HIV-1, Human immunodeficiency virus-1; SARS-CoV-2, Severe acute respiratory syndrome coronavirus-2; YFV, Yellow fever virus; CVB3, Coxsackievirus B3; KSHV, Kaposi’s sarcoma-associated herpesvirus; STAs, statins; LOV, lovastatin; FLV, fluvastatin; PRV, pravastatin; ATV, atorvastatin; SIM, simvastatin; RSV, rosuvastatin; CRV, cerivastatin; MEV, mevastatin; PTV, Pitavastatin; EZE, Ezetimibe; MET, metformin; SVZ, Subventricular zone; SVR Sustained virological response; DHF, Dengue hemorrhagic fever; DSS, Shock syndrome; SD, Severe dengue; cccDNA, Circular covalently closed DNA.*

The use of STAs has been associated with a lower risk of mortality among people with COVID-19 (Zhang et al., 2020; Torres-Peña et al., 2021) and Influenza virus (Kwong et al., 2009); this effect has been related to their immunomodulatory properties (Fedson, 2013; Gorabi et al., 2020). However, its use to treat these diseases remains controversial (Hui et al., 2018; Atamna et al., 2019; Lima Martínez et al., 2020; Subir et al., 2020; Zhao and Peng, 2021). The importance of lipids during coronavirus infection is poorly explored; however, a remodeling of lipid metabolism has been described during the characterization of the lipidomic profile of human coronavirus-infected cells (Yan et al., 2019). It also has been described that aberrant lipid metabolism in morbidly obese individuals adversely affects the COVID-19 immune response and increases disease severity (Kindrachuk et al., 2015). This evidence suggests that lipids play an essential role in the pathogenesis and viral cycle of SARS-CoV-2.

Similar to DENV, it has been reported that cholesterol is required for stability and infectivity of IAV and respiratory syncytial virus (RSV). Therefore, cholesterol depletion by STAs decreases both viruses’ infectivity and viral production (Gower and Graham, 2001; San-Juan-Vergara et al., 2012; Bajimaya et al., 2017). Interestingly, RSV replication is restored by mevalonolactone, which salvages the cholesterol biosynthetic pathway, indicating that the effect of STAs on RSV replication is mediated by the products of this pathway and not by alternative mechanisms (Gower and Graham, 2001).

STAs can also significantly reduce levels of proinflammatory cytokines in RSV-infected cells (Ravi et al., 2013). For the IAV, the benefits of treatment are strongly linked to immunoregulatory effects and not to impact on viral replication (Liu et al., 2009; Peng et al., 2014; Hui et al., 2018).

It has also been documented that human immunodeficiency virus type 1 (HIV-1) and HCV are sensitive to statin-mediated cholesterol-lowering, which affects viral entry, fusion, and replication processes (Mañes et al., 2000; Liao et al., 2003; del Real et al., 2004; Bley et al., 2020). STAs decreased viral load and increased CD4 + cell counts in acute infection models and in chronically HIV-1-infected patients; this effect was blocked by adding l-mevalonate or GGP, but not by cholesterol (del Real et al., 2004). Additionally, STAs can also interfere with the prenylation of proteins required for HIV and RSV (Gower and Graham, 2001; del Real et al., 2004).

STAs were also suggested as a possible adjunctive therapy for Ebola virus disease (EVD) to counteract the inflammation and immune system dysregulation caused by the Ebola virus (EBOV) (Fedson et al., 2015). *In vitro* assays have shown that STAs can decrease infection and reduce the infectivity of the EBOV (Shrivastava-Ranjan et al., 2018). Interestingly, when different inhibitors of the mevalonate pathway were tested, no antiviral effect was observed when inhibiting HMGCR, but it was observed when inhibiting FPPS, Farnesyl pyrophosphate synthase, and OSC, 2,3 oxidosqualene cyclase (Shoemaker et al., 2013). Lipids, such as phosphatidylserine, are critical for the EBOV replicative cycle (Soni and Stahelin, 2014), but the role of cholesterol during EBOV infection is still being studied. The existence of cholesterol-dependent interactions between EBOV glycoproteins (GPs) suggests that cholesterol is critical for viral assembly and the pathology caused by EBOV (Hacke et al., 2015). In this regard, STAs can suppress EBOV infectivity by interfering with glycoprotein processing, and this inhibition can be reversed by the exogenous mevalonate (Shrivastava-Ranjan et al., 2018).

Interestingly, as occurred with EBOV, STAs inhibited hepatitis B virus (HBV), and the addition of mevalonate abolished the anti-HBV effect, suggesting that the mevalonate pathway could be a therapeutic target for all enveloped viruses (Bader and Korba, 2010). However, it should be noted that only a few studies have tested the antiviral effect of STAs against DNA viruses, and the evidence is inconsistent (Potena et al., 2004; Bader and Korba, 2010; Wu, 2013). On the other hand, although the antiviral effect of statins against enveloped RNA viruses is apparently a common factor, it is clear that their efficacy will depend directly on the characteristics of each virus and the pathology it causes in the host.

The EZE is another cholesterol-lowering drug that, unlike STAs, has been little studied for its antiviral effect. There is only one report showing the cholesterol-dependent anti-DENV effect of this drug to our knowledge (Osuna-Ramos et al., 2018a). Although it has been shown that this drug can counteract HCV, HBV, and EBOV infection, this effect seems to be related to the blockade of the viral entry receptor and not to the hypolipidemic effect of this drug (Carette et al., 2011; Sainz et al., 2012; Lucifora et al., 2013; Herbert et al., 2015). Therefore, further studies are required to elucidate the antiviral effects of EZE.

Finally, metformin, which in 1940 was used to treat influenza (Bailey, 2017), is another drug with lipid-lowering effects that has gained interest in recent decades due to its pleiotropic effects and its antiviral properties (Ibrahim et al., 2021). As described above, some viruses can modulate AMPK protein activity during infection to their advantage (Bhutta et al., 2021). The HCV (Mankouri et al., 2010; Yu et al., 2013), Epstein-Bar virus (EBV) (Lo et al., 2013), and DENV (Soto-Acosta et al., 2017) can downregulate the active form of AMPK to obtain a favorable lipid microenvironment and are therefore sensitive to AMPK activators. However, besides DENV and ZIKV, few studies have tested the antiviral effects of MET.

*In vitro* assays have shown that MET restricts coxsackievirus B3 (CVB3) replication by inhibiting lipid accumulation. CVB3 manipulates and modifies cellular lipid metabolism to enhance viral replication; therefore, activation of AMPK by MET restricts infection by inhibiting lipid synthesis-associated gene expression (Xie et al., 2015). It has also been described that activation of AMPK by MET restricts Kaposi’s sarcoma-associated herpesvirus (KSHV), a DNA virus, by inhibiting viral lytic gene expression and the production of infectious virions (Cheng et al., 2016). HCV is also sensitive to MET treatments, which enhance the innate immune response in hepatocarcinoma cells (Tsai et al., 2017). In addition, in patients with chronic hepatitis C, the use of MET in combination with other antivirals has been associated with a better sustained virological response (Yu et al., 2012).

It has also been suggested that the immunomodulatory and immunometabolic effects of MET provide benefits in the treatment of patients with type 2 diabetes and insulin resistance during Covid-19 disease (Singh and Singh, 2020; Hashemi and Pezeshki, 2021; O’Carroll and O’Neill, 2021). Retrospective studies have suggested a relative survival benefit in SARS-CoV-2 infected elderly persons taking MET compared to those not taking MET (Luo P. et al., 2020, p. 19; Ghany et al., 2021; Lally et al., 2021, p. 2).

Although the antiviral mechanisms are unknown, this could be attributed to its anti-inflammatory potential (O’Carroll and O’Neill, 2021, p. 19). Because complex lipid membrane formation and palmitoylation of coronavirus proteins are essential during viral replication and assembly, it has been suggested that FASN inhibition and AMPK activation could prevent coronavirus replication. This is based on *in vitro* findings and clinical data revealing that the FASN inhibitor, orlistat, and the AMPK activator, MET, can inhibit coronavirus replication and reduce systemic inflammation to restore immune homeostasis (Tanner and Alfieri, 2021, p. 19). On the other hand, AMPK activation by MET could act on the PI3K/AKT/mTOR pathway, an essential pathway in MERS-CoV infection (Kindrachuk et al., 2015).

All the results described above indicate that the antiviral effect of lipid-lowering drugs such as STAs and MET is based on their lipid-lowering properties and the pleiotropic properties they offer. Therefore they could be a viable alternative for treating viral infections, specifically for viruses with high lipid demand.

## Conclusion

Evidence suggests that ZIKV and DENV have adapted to and co-evolved with cellular lipid metabolism to enhance their replication. Because of the close link between the viral cycle and cell lipid metabolism, FDA-approved drugs with lipid-lowering effects have been considered as potential host-directed therapies to inhibit viral replication. *In vitro* assays have shown that STAs, EZE, and MET effectively inhibit ZIKV and DENV. However, there are limited *in vivo* and clinical trials demonstrating the effectiveness of these drugs during flavivirus infections, so further studies are needed to determine their antiviral effect. Overall, the evidence suggests that AMPK protein is a therapeutic target for DENV and not for ZIKV. Therefore, MET could be safe and efficient for treating DENV infection.

In contrast, the use of STAs or drugs that directly inhibit key enzymes of the mevalonate and/or fatty acid pathway might be a better strategy for ZIKV. Furthermore, the combination with EZE, which inhibits cholesterol uptake, could offer effective therapy for inhibiting these flaviviruses. Hence, further studies are essential to determine whether the use of lipid-lowering drugs, either in single doses or in combination, is feasible for treating DENV and ZIKV infection.

## Author Contributions

CF-M, CC-R, and JR-R wrote the manuscript. AH-M, JO-R, LD, and RA revised the draft version of the manuscript. AG-G and SP-R prepared the figure and figure legend. RA conceived the idea review. All authors critically reviewed the final version of the manuscript.

## Conflict of Interest

The authors declare that the research was conducted in the absence of any commercial or financial relationships that could be construed as a potential conflict of interest.

## Publisher’s Note

All claims expressed in this article are solely those of the authors and do not necessarily represent those of their affiliated organizations, or those of the publisher, the editors and the reviewers. Any product that may be evaluated in this article, or claim that may be made by its manufacturer, is not guaranteed or endorsed by the publisher.
